# RSV prophylaxis use in high-risk infants in Western Australia, 2002-2013: a record linkage cohort study

**DOI:** 10.1186/s12887-020-02390-5

**Published:** 2020-10-22

**Authors:** Ruomei Xu, Parveen Fathima, Tobias Strunk, Nicholas de Klerk, Thomas L. Snelling, Peter C. Richmond, Anthony D. Keil, Hannah C. Moore

**Affiliations:** 1 Wesfarmers Centre of Vaccines and Infectious Diseases, Telethon Kids Institute, University of Western Australia, PO Box 855, Crawley, WA 6872 Australia; 2 Neonatal Directorate, King Edward Memorial Hospital, Perth, WA Australia; 3 Departments of Immunology and General Paediatrics, Perth Children’s Hospital, Perth, WA Australia; 4 Menzies School of Health Research, Charles Darwin University, Casuarina, NT Australia; 5 School of Public Health, Curtin University, Perth, WA Australia; 6 Division of Paediatrics, School of Medicine, University of Western Australia, Perth, WA Australia; 7 PathWest Laboratory Medicine WA, Perth, WA Australia

**Keywords:** Respiratory syncytial virus, Palivizumab, Infants, Neonatal intensive care unit, High-risk

## Abstract

**Background:**

The monoclonal antibody, palivizumab is licensed for use in high-risk infants to prevent severe illness caused by respiratory syncytial virus (RSV). The level of its use and compliance with current jurisdictional guidelines which were amended in 2010, is unknown. We determined the level of palivizumab use in a cohort of high-risk infants in Western Australia.

**Methods:**

Using probabilistically linked administrative data, we conducted a birth cohort study within tertiary neonatal intensive care units (NICUs) born between 2002 and 2013. We described palivizumab use by patient characteristics, eligibility criteria according to guidelines over the period of study and identified predictors of its use.

**Results:**

Of 24,329 infants admitted to tertiary NICUs, 271 (1.1%) were dispensed 744 palivizumab doses with 62.5% being dispensed to infants born 2010–2013. The median number of doses received was 2. A total of 2679 infants met at least one of three criteria for palivizumab (criteria 1: gestational age at birth < 28 weeks and chronic lung disease; criteria 2: gestational age < 28 weeks and Aboriginal; criteria 3: congenital heart disease not otherwise in criteria 1 or 2). The extent of palivizumab use differed across the 3 groups. Of 803 infants meeting criteria 1, 21.8% received at least 1 dose of palivizumab; 52.8% from 2010 onwards. From 174 infants meeting criteria 2, 14.4% received at least 1 dose; 43.1% from 2010 onwards and from 1804 births meeting criteria 3, only 3.7% received at least 1 dose; 5.4% from year of birth 2010 onwards). In adjusted analyses, being born after 2010, being extreme preterm, chronic lung disease, congenital lung disease and being born in autumn or winter were independent predictors of palivizumab use.

**Conclusion:**

In this high-risk setting and notwithstanding the limitations of our data sources, the level of compliance of palivizumab use against current guidelines was low. Most doses were dispensed to infants meeting at least one high-risk criterion. Evidence of incomplete dosing is an important finding in light of recent developments of single dose monoclonal antibodies offering longer protection.

## Background

Respiratory syncytial virus (RSV) is a leading cause of acute lower respiratory infection (ALRI) in young children [1]. In 2015, the annual global burden of RSV-related ALRI was estimated to be 33.1 million infection episodes, resulting in 3.2 million hospitalisations and 59,600 deaths in children younger than five years [2]. Infants born preterm and those with congenital heart disease (CHD) or chronic lung disease of prematurity (CLD), are at high risk for severe RSV [3, 4]. Children aged < 6 months are also more likely to have severe RSV compared with older children [5].

Although RSV-associated mortality in Australia is low, RSV remains a leading cause of hospitalisation, surpassing that of current vaccine-preventable disease such as influenza and rotavirus [6]. In Western Australia (WA), where we have the ability to link routinely collected laboratory data to perinatal and hospitalisation data, RSV is the most frequently identified virus among children hospitalised due to ALRI with seasonal peaks in the Southern Hemisphere winter months of June–August [7].

While a number of vaccine candidates are in clinical trials, there is currently no licensed vaccine targeting RSV [8, 9]. At present, the only available strategy for prevention of RSV infection is the administration of palivizumab, an injectable monoclonal antibody [10]. In Australia, palivizumab was licensed by the Therapeutic Goods Administration in 1999 for use in high-risk infants [11]. However, there are no nationally agreed guidelines for its use. WA is unique among Australian jurisdictions in funding palivizumab for infants less than 12 months old with CHD or requiring home oxygen for CLD in their first RSV season [12]. Since 2010, one of two tertiary neonatal intensive care units (NICU) in WA has also used palivizumab for in-hospital prophylaxis to prevent nosocomial RSV infection [13]. Palivizumab administration requires monthly intramuscular injections in order to achieve and maintain a protective concentration of antibodies [10]. Five monthly doses of palivizumab are recommended during an RSV season [10]. Palivizumab is an expensive prevention strategy costing approximately $AUD 8750 per patient; hence its use is limited [12].

To address the lack of data regarding the level of palivizumab use in Australia with the extended guidelines, we aimed to describe the extent of palivizumab use in a high-risk cohort of WA-born infants up to age 2 years and to investigate the demographic and clinical factors associated with its use.

## Methods

### Setting

WA is the largest state in Australia by area, covering approximately 2.5 million square kilometres, and in 2016 had an estimated population of 2.5 million [14]. Newborns requiring admission to NICUs account for approximately 2.6% of all live births in Australia [15]. In WA, there are two tertiary level NICUs, at the Princess Margaret Hospital for Children (now Perth Children’s Hospital) and at the King Edward Memorial Hospital for Women.

### Study design and study population

We conducted a retrospective record linkage cohort study. The cohort was defined as infants born in WA between 1 January 2002 and 31 December 2013 inclusive, and admitted to either of the two tertiary NICUs.

### Data sources

**Fig. 1:**
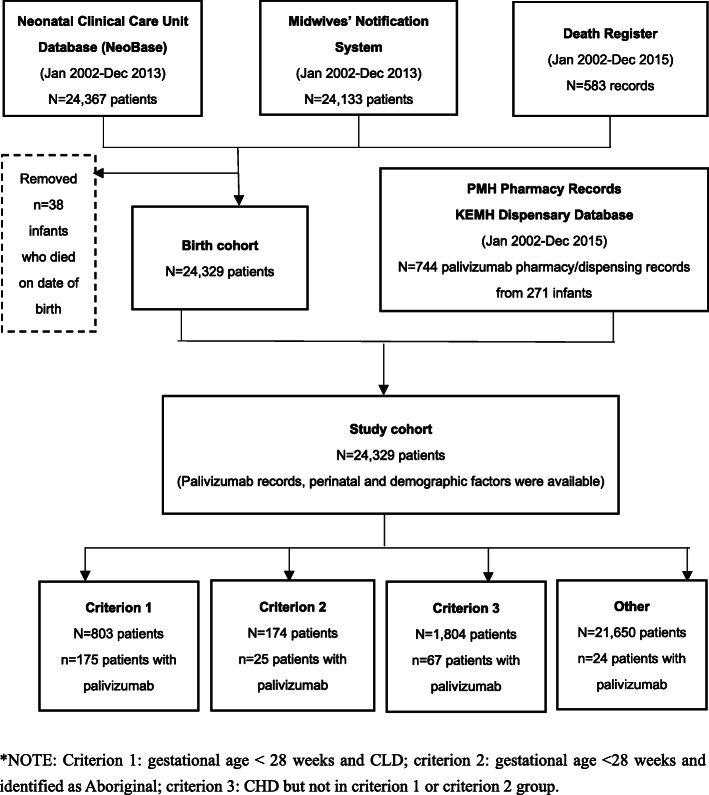
Summary of datasets used

The cohort was identified using the Neonatal Clinical Care Unit Database (NeoBase, 2002–2013), the WA Midwives’ Notification System (2002–2013), and the WA Death Register (2002–2015). Data on palivizumab use relating to the cohort children were extracted, from several historical and current pharmacy records and dispensing datasets (2002–2015, only available for the first 2 years of life), and probabilistically linked by the Western Australia Data Linkage Branch using a set of unique person identifiers (Fig. 1) [16]. This analysis was part of a larger project that also included linked information on hospitalisations identified from the Hospital Morbidity Data Collection.

### Variables of interest

The primary variable of interest was receipt of palivizumab, by the number of doses, as recorded across multiple dispensary and pharmacy datasets. In the absence of date of administration, we used the date of dispensing. Maternal- and infant-related demographic and perinatal factors of interest for children in the cohort were identified from the electronic neonatal database and the Midwives’ Notification System. Infant-related factors included: sex, year of birth, Aboriginal and/or Torres Strait Islander (herein referred to as Aboriginal) status as identified through a validated algorithm, [17] gestational age, birth weight, presence/absence of major congenital anomalies, CHD, CLD, multiple birth and season of birth (spring [September–November], summer [December–February], autumn [March–May], winter [June–August]). Maternal factors included socioeconomic status, maternal smoking during pregnancy and the number of previous pregnancies. CHD was defined using all International Classification of Diseases version 10 Australian modification (ICD-10 AM) diagnosis codes in the hospitalisation dataset (Q20–26) which includes those that would identify hemodynamically significant heart disease as well as ventricular and atrial septal defects and patent ductus arteriosis. CLD was defined using an indicator variable on the neonatal dataset (for oxygen requirement after 36 weeks of postmenstrual age) as well as all ICD-10 AM diagnosis codes in the hospitalisation dataset (P27.1). Socioeconomic status was defined using Socio-Economic Indexes for Area, [18] divided into five categories ranging from the most (0–10%) to least disadvantaged (90–100%). Based on the maternal postcode at the time of delivery, geographical location of residence was defined as metropolitan (north metropolitan and south metropolitan), rural (South West, Great Southern, Midwest and Wheatbelt) or remote (Kimberley, Pilbara, Goldfields). The RSV season was defined as being from April to October in any given year [19].

### Statistical analysis

We assessed the actual use of palivizumab in the cohort against the indications given in the guidelines used at the two NICUs. These indications were: gestational age < 28 weeks and CLD (defined as criterion 1); gestational age < 28 weeks and identified as Aboriginal (criterion 2); and CHD (criterion 3). Due to the overlap between criteria, we only counted infants as fulfilling criterion 3 if they did not also meet either of the first two eligibility criteria. A small number of infants met both criterion 1 and criterion 2. Infants who did not fit any of the three eligibility criteria were classified as “other”.

We assessed the proportion of infants receiving at least one dose of palivizumab throughout the study period in the overall cohort as well as the number of doses received in the infants’ first two years of life to document the total number of palivizumab doses received. We compared the level of palivizumab use between infants across the different eligibility criteria and across different perinatal and demographic factors. To assess the predictors of palivizumab use, univariable and multiple logistic regression models reporting odds ratios (ORs) with 95% confidence intervals (CIs) were used. Variables not associated with use in the univariable models (*p* ≥ 0.2) were excluded from multiple regression analysis unless the variables were part of the eligibility criteria for palivizumab use. EpiBasic (version 3) was used to calculate exact 95% CIs. Other analyses were performed using STATA (version 14.1). At the request of the data custodians, individual cell sizes in the reported tables less than 5 have been suppressed.

## Results

The cohort comprised 24,329 infants born in WA between 1 January 2002 and 31 December 2013; 38 infants who died on their day of birth were excluded. Of the infants in the cohort, 13,437 (55.2%) were male and 3005 (12.4%) were Aboriginal.

**Fig. 2:**
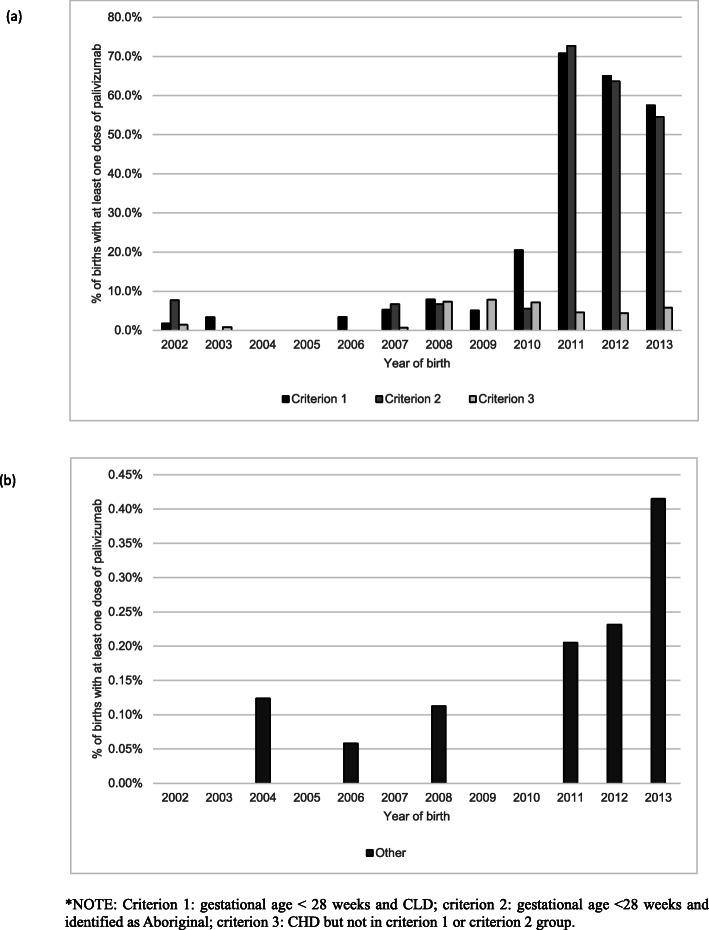
Proportion of infants receiving at least one dose of palivizumab by **a** eligibility criteria and **b** those not meeting the eligibility criteria. Note the differences in scale between **a** and **b**

A total of 271 (1.1%) of infants in the cohort received a total of 744 doses of palivizumab between 2002 and 2015. The proportion of infants receiving at least one dose of palivizumab increased after 2010 (Fig. 2), resulting in more than half (62.5% [*n* = 465]) of the doses being dispensed to infants born between 2010 and 2013.

Of the infants in the cohort, 2679 (11.0%) met at least one of the three eligibility criteria for palivizumab use. Of these, only 247 (9.2%) of infants received at least one dose of palivizumab. Of the infants fitting the eligibility criteria, 8.3 (30.0%) met criterion 1 (< 28 weeks gestation and CLD), 174 (6.5%) met criterion 2 (< 28 weeks and Aboriginal), and 1804 (67.3%) met criterion 3 (CHD but not in criterion 1 or criterion 2). There were 102 (3.8%) of infants who fulfilled both criterion 1 and criterion 2, and they were reported in both categories. The extent of palivizumab use differed across the eligibility criteria groups. Criterion 1 had the largest proportion of infants with at least 1 dose of palivizumab (21.8%; 52.8% from year of birth 2010 onwards), followed by criterion 2 (14.4%; 43.1% from year of birth 2010 onwards) and criterion 3 (3.7%; 5.4% from year of birth 2010 onwards). Of those 67 infants in criterion 3 who received at least 1 dose of palivizumab, 27 (40.2%) had a severe CHD (being either malformation of cardiac chambers and connections [Q20], or hypoplastic right heart syndrome [Q22.6] or atresia of pulmonary artery [Q25.5]) and 40 (59.7%) had a ventricular or atrial septal defect or patent ductus arteriosus (Q21.0, Q21.1 or Q25.0). There were 20 infants with documented palivizumab use who met both criterion 1 and criterion 2. A total of 21,650 infants in the cohort did not meet any of the eligibility criteria for palivizumab. However, 24 (0.1%) of these infants received at least 1 dose.

Table 1 shows the perinatal and demographic characteristics of infants receiving palivizumab by the eligibility criteria. The majority of palivizumab recipients meeting criterion 1 (89.7% [*n* = 157]) or criterion 2 (72.0% [*n* = 18]) had CHD. Of infants meeting either criterion1 or 2 but did not receive palivizumab, 63 (76.8%) had CHD. Similarly, 20 (80.0%) of infants receiving palivizumab in criterion 2 group had CLD. Most infants who met the eligibility criteria and received palivizumab were born in autumn or winter. The proportions of infants with palivizumab across different perinatal and demographic factors are shown in Table 2. Overall, 33 (12.2%) of palivizumab recipients were Aboriginal, 208 (76.8%) were born < 28 weeks gestation, 226 (83.4%) had CHD, and 192 (70.8%) had CLD. In univariable analyses, extreme preterm birth, CLD and CHD were most strongly associated with palivizumab use in the overall cohort (Table 2). With the exception of sex, all factors were included in the multivariable model. After adjusting for other factors, extreme preterm birth (adjusted OR [aOR] 7.4; 95% CI: 1.8–30.9), being born after 2010 (aOR 8.8; 95% CI: 6.1–12.6), having CHD (aOR 5.8; 95% CI: 3.8–8.9) or a major congenital anomaly (aOR 3.8; 95% CI: 2.7–5.4) and being born in autumn (aOR 4.2; 95%CI: 2.6–6.7) or winter (aOR 3.4; 95% CI: 2.1–5.6) were associated with palivizumab receipt, but very low birth weight (< 1500 g) and being born in rural regions were not (Table 2). Being Aboriginal was not a predictor of palivizumab receipt (aOR 0.8; 95% CI: 0.4–1.4; Table 2).

**Table 1:** Characteristics of infants receiving at least one dose of palivizumab, by eligibility criteria

Characteristic	Infants receiving palivizumab							
	Criterion 1 (*N* = 175/803)		Criterion 2 (*N* = 25/174)		Criterion 3 (*N* = 67/1804)		Other (*N* = 24/21,650)	
	*n*	(%)	*n*	(%)	*n*	(%)	*n*	(%)
Sex								
Males	94	(53.7)	17	(68.0)	44	(65.7)	16	(66.7)
Females	81	(46.3)	8	(32.0)	23	(34.3)	8	(33.3)
Year of birth								
2002–2009	17	(9.7)	< 5	(< 20.0)	30	(44.8)	5	(20.8)
2010–2013	158	(90.3)	< 25	(< 100.0)	37	(55.2)	19	(79.2)
Indigenous status								
Aboriginal	20	(11.4)	25	(100.0)	6	(9.0)	< 5	(< 20.8)
Non-Aboriginal	155	(88.6)	0	–	61	(91.0)	< 24	(< 100.0)
Gestational age								
< 28 weeks	175	(100.0)	25	(100.0)	12	(17.9)	16	(66.7)
28–32 weeks	0	–	0	–	13	(19.4)	< 5	(< 20.8)
33–36 weeks	0	–	0	–	12	(17.9)	< 5	(< 20.8)
≥ 37 weeks	0	–	0	–	30	(44.8)	5	(20.8)
Birth weight								
< 1500 g	175	(100.0)	25	(100.0)	24	(35.8)	17	(70.8)
1500–2499 g	0	–	0	–	13	(19.4)	< 5	(< 20.8)
2500–2999 g	0	–	0	–	11	(16.4)	< 5	(< 20.8)
3000–3499 g	0	–	0	–	11	(16.4)	< 5	(< 20.8)
≥ 3500 g	0	–	0	–	8	(11.9)	< 5	(< 20.8)
Geographical area of birth*								
Metropolitan	125	(71.4)	< 5	(< 20.0)	52	(77.6)	18	(75.0)
Rural	35	(20.0)	< 10	(< 40.0)	< 15	(< 22.4)	< 10	(< 41.7)
Remote	14	(8.0)	12	(48.0)	< 5	(< 7.5)	< 5	(< 20.8)
Major congenital anomalies								
Yes	77	(44.0)	16	(64.0)	40	(59.7)	7	(29.2)
No	98	(56.0)	9	(36.0)	27	(40.3)	17	(70.8)
Congenital heart disease								
Yes	157	(89.7)	18	(72.0)	67	(100.0)	0	–
No	18	(10.3)	7	(28.0)	0	–	24	(100.0)
Chronic lung disease								
Yes	175	(100.0)	20	(80.0)	15	(22.4)	< 5	(< 20.8)
No	0	–	5	(20.0)	52	(77.6)	< 24	(< 100.0)
Season of birth								
Spring	23	(13.1)	< 5	(< 20.0)	13	(19.4)	< 5	(< 20.8)
Summer	30	(17.1)	< 5	(< 20.0)	12	(17.9)	< 5	(< 20.8)
Autumn	66	(37.7)	10	(40.0)	24	(35.8)	10	(41.7)
Winter	56	(32.0)	9	(36.0)	18	(26.9)	11	(45.8)
Multiple birth								
Singleton	135	(77.1)	< 25	(< 100.0)	60	(89.6)	< 24	(< 100.0)
Multiple	40	(22.9)	< 5	(< 20.0)	7	(10.4)	< 5	(< 20.8)
Number of previous pregnancies								
0	62	(35.4)	9	(36.0)	18	(26.9)	8	(33.3)
1	41	(23.4)	< 5	(< 20.0)	23	(34.3)	6	(25.0)
2	19	(10.9)	< 5	(< 20.0)	8	(11.9)	< 10	(< 41.7)
≥ 3	53	(30.3)	11	(44.0)	18	(26.9)	< 5	(< 20.8)
Socioeconomic status								
0–10% (most dis)	29	(16.6)	7	(28.0)	9	(13.4)	< 5	(< 20.8)
10–25%	18	(10.3)	< 10	(< 40.0)	10	(14.9)	7	(29.2)
25–75%	96	(54.9)	< 5	(< 20.0)	28	(41.8)	9	(37.5)
75–90%	13	(7.4)	0	–	< 15	(< 22.4)	< 5	(< 20.8)
90–100%	8	(4.6)	0	–	< 5	(< 7.5)	< 5	(< 20.8)
Missing	11	(6.3)	7	(28.0)	0	–	0	–
Mother smoking during pregnancy								
Yes	29	(16.6)	13	(52.0)	9	(13.4)	5	(20.8)
No	146	(83.4)	12	(48.0)	58	(86.6)	19	(79.2)

*Missing for 1 infant receiving palivizumab

*NOTE: (1) Cell sizes of less than 5 have been suppressed

(2) Criterion 1: gestational age < 28 weeks and CLD; criterion 2: gestational age < 28 weeks and identified as Aboriginal; criterion 3: CHD but not in criterion 1 or criterion 2 group

**Table 2:** Proportion and odds ratio of receipt of at least one dose of palivizumab

Characteristic	Infants receiving at least 1 dose of palivizumab *N* = 271		Infants receiving no palivizumab *N* = 24,058		Univariable		Multivariable	
	*n*	(%)	*n*	(%)	OR	(95% CI)	aOR	(95% CI)
Sex								
Males	157	(57.9)	13,280	(55.2)	Ref		Ref	
Females	114	(42.1)	10,776	(44.8)	0.9	(0.7–1.1)	–	–
Year of birth								
2002–2009	52	(19.2)	14,844	(61.7)	Ref		Ref	
2010–2013	219	(80.8)	9214	(38.3)	6.8	(5.0–9.2)	8.8	(6.1–12.6)
Indigenous status								
Non-Aboriginal	238	(87.8)	21,086	(87.6)	Ref		Ref	
Aboriginal	33	(12.2)	2972	(12.4)	1.0	(0.7–1.4)	0.8	(0.4–1.4)
Gestational age								
< 28 weeks	208	(76.8)	1007	(4.2)	66.4	(46.2–95.6)	7.4	(1.8–30.9)
28–32 weeks	15	(5.5)	3688	(15.3)	1.3	(0.7–2.4)	0.5	(0.1–1.9)
33–36 weeks	13	(4.8)	8107	(33.7)	0.5	(0.3–1.0)	0.5	(0.2–1.2)
≥ 37 weeks	35	(12.9)	11,256	(46.8)	Ref		Ref	
Birth weight								
< 1500 g	221	(81.5)	2926	(12.2)	22.3	(13.0–38.3)	1.5	(0.4–6.3)
1500–2499 g	14	(5.2)	7786	(32.4)	0.5	(0.3–1.1)	0.9	(0.4–2.1)
2500–2999 g	14	(5.2)	4132	(17.2)	Ref		Ref	
3000–3499 g	13	(4.8)	4341	(18.0)	0.9	(0.4–1.9)	0.6	(0.3–1.4)
≥ 3500 g	9	(3.3)	4873	(20.3)	0.6	(0.2–1.3)	0.4	(0.2–1.0)
Geographical area of birth								
Metropolitan	196	(72.3)	19,010	(79.0)	Ref		Ref	
Rural	55	(20.3)	3262	(13.6)	1.6	(1.2–2.2)	1.6	(1.0–2.5)
Remote	19	(7.0)	1701	(7.1)	1.1	(0.7–1.7)	0.9	(0.4–1.9)
Major congenital anomalies								
No	145	(53.5)	22,061	(91.7)	Ref		Ref	
Yes	126	(46.5)	1997	(8.3)	9.6	(7.5–12.2)	3.8	(2.7–5.4)
Congenital heart disease								
No	45	(16.6)	21,709	(90.2)	Ref		Ref	
Yes	226	(83.4)	2259	(9.4)	48.3	(35.0–66.7)	5.8	(3.8–8.9)
Chronic lung disease								
No	79	(29.2)	23,111	(96.1)	Ref		Ref	
Yes	192	(70.8)	947	(3.9)	59.3	(45.3–77.7)	2.8	(1.8–4.5)
Season of birth								
Spring	38	(14.0)	5918	(24.6)	Ref		Ref	
Summer	43	(15.9)	6005	(25.0)	1.1	(0.7–1.7)	1.1	(0.6–1.9)
Autumn	104	(38.4)	6170	(25.6)	2.6	(1.8–3.8)	4.2	(2.6–6.7)
Winter	86	(31.7)	5965	(24.8)	2.3	(1.5–3.3)	3.4	(2.1–5.6)
Multiple birth								
Singleton	221	(81.5)	20,527	(85.3)	Ref		Ref	
Multiple	50	(18.5)	3495	(14.5)	1.3	(1.0–1.8)	0.6	(0.4–0.9)
Number of previous pregnancies								
0	90	(33.2)	7595	(31.6)	Ref		Ref	
1	70	(25.8)	6272	(26.1)	0.9	(0.7–1.3)	1.3	(0.9–1.9)
2	33	(12.2)	4044	(16.8)	0.7	(0.5–1.0)	1.1	(0.7–1.9)
≥ 3	78	(28.8)	6111	(25.4)	1.1	(0.8–1.5)	1.4	(0.9–2.1)
Socioeconomic status								
0–10% (most dis)	42	(15.5)	2967	(12.3)	Ref		Ref	
10–25%	38	(14.0)	4628	(19.2)	0.6	(0.4–0.9)	0.6	(0.4–1.1)
25–75%	134	(49.4)	10,688	(44.4)	0.9	(0.6–1.3)	0.8	(0.5–1.2)
75–90%	28	(10.3)	2780	(11.6)	0.7	(0.4–1.2)	0.8	(0.5–1.6)
90–100%	12	(4.4)	1213	(5.0)	0.7	(0.4–1.3)	0.7	(0.3–1.5)
Mother smoking during pregnancy								
No	224	(82.7)	18,861	(78.4)	Ref		Ref	
Yes	47	(17.3)	5161	(21.5)	0.8	(0.6–1.1)	1.0	(0.6–1.5)

All palivizumab doses were dispensed between March and October. The median number of palivizumab doses received was 2 (range: 1–15; Table 3). As shown in Tables 4, 562 (75.5%) of palivizumab use was in infant’s first RSV season with a further 147 (19.8%) of doses dispensed in their second RSV season. A small number of infants in this cohort received palivizumab in their third RSV season. Of all infants receiving palivizumab in their first RSV season, 79 (32.8%) only received one dose, 81 (33.6%) received 2 doses and 44 (18.3%) received 3 doses. After restricting to those born before June in their first RSV season (to allow the full completion of a 5-monthly course over their first RSV season), 82.0% of infants only received 3 or less doses with only 14 (10.5%) of infants receiving at least 5 doses.

**Table 3:** Median age at the first, second, third, fourth and fifth dose of palivizumab dispensing

Dose	Number of infants	Median age in days (IQR)
1st	271	39 (33–111)
2nd	191	67 (62–142)
3rd	106	96 (90–251)
4th	62	266 (121–372)
5th	47	318 (154–417)

*IQR inter-quartile range

**Table 4:** Number of palivizumab doses dispensed by RSV season and eligibility criteria

	Criterion 1 *N* = 457		Criterion 2 *N* = 68		Criterion 3 *N* = 227		Other *N* = 50		Total *N* = 744	
	*n*	(%)	*n*	(%)	*n*	(%)	*n*	(%)	*n*	(%)
First RSV season	366	(80.1)	57	(83.8)	150	(66.1)	36	(72.0)	562	(75.5)
Second RSV season	75	(16.4)	< 10	(< 14.7)	70	(30.8)	< 5	(< 10.0)	147	(19.8)
Third RSV season	16	(3.5)	< 5	(< 7.4)	7	(3.1)	< 15	(< 30.0)	35	(4.7)

*NOTE: (1) Cell sizes of less than 5 have been suppressed

(2) Criterion 1: gestational age < 28 weeks and CLD; criterion 2: gestational age < 28 weeks and identified as Aboriginal; criterion 3: CHD but not in criterion 1 or criterion 2 group

## Discussion

We present here real-world data on the use of palivizumab in a high-risk cohort of infants in Western Australia. More than 90 % of infants receiving palivizumab had at least one high-risk condition for palivizumab use; a study conducted in New South Wales reported only 67.5% of infants administered palivizumab met their local hospital guidelines [20]. We also found that gestational age < 28 weeks, CHD and being born after 2010 were the three strongest independent predictors of palivizumab use in the cohort.

The increased use of palivizumab between 2010 and 2013 reflected the impact of the neonatal medication protocol established at King Edward Memorial Hospital in 2010 [13]. These guidelines were established following consequential episodes (including deaths) of nosocomial RSV transmission. Despite these recommendations and extended guidelines, we found that only 9.2% (*n* = 247) of those meeting the eligibility criteria received at least one dose of palivizumab.

Our study found that few children received all five recommended doses of palivizumab. Even after restricting to those infants born before the middle of the RSV season to allow a full course of palivizumab over the season, 82.0% of the recipients received three or fewer doses, suggesting compliance with recommendations was low. However, for those infants with CLD, cessation of prophylaxis most likely coincides with discontinuation of supplementary home oxygen (Dr Wilson, personal communication).

The association between palivizumab non-compliance and increased rates of RSV-related hospitalisations had been demonstrated previously [21]. In an American study, young children who received incomplete palivizumab prophylaxis had around 3-fold higher risk for RSV-related hospitalisations compared with children receiving all recommended doses [22]. It is also believed that factors such as low birth weight can affect adherence to palivizumab dosing recommendations [22]. It is important to improve adherence among infants receiving palivizumab in order to maximise the protection. A previous study suggested that receiving palivizumab at home instead of in a paediatrician’s office could not only contribute to better adherence but also lead to a greater parental satisfaction [23]. The relationship between adherence with the recommended dosing schedule and the effectiveness of reducing RSV-hospitalisations now needs to be explored.

Not surprisingly, the factors with the strongest association with palivizumab use included indications in the current eligibility criteria. However, being Aboriginal (part of criteria 2) was not an independent predictor. Reasons for incomplete palivizumab use among eligible Aboriginal infants could include the failure to correctly identify infants as Aboriginal, or a higher proportion of these infants being from remote and rural areas where access to palivizumab is more problematic; however, birth in a rural location was not found to be a predictor of palivizumab use. Aboriginal children experience hospitalisation rates for ALRI 7.5 times higher than non-Aboriginal children, [24] and RSV-confirmed hospitalisation rates are approximately 2 times higher in Aboriginal than non-Aboriginal children (Moore, unpublished data). The effectiveness of palivizumab in Aboriginal children now needs to be determined.

The major strength of our study is the use of population-based datasets, with near complete perinatal and demographic information to form the study cohort. Despite this, our study does have several limitations which should be considered when interpreting the results. Firstly, we did not have data on palivizumab administration, so we assumed all the dispensed doses were administered, and that the date of palivizumab dispensing was the same as the date of administration. Therefore, the data extracted from the dispensary datasets might not accurately reflect the timing of palivizumab administration in the cohort. Secondly, palivizumab data were collected from several historical and current databases. Some of the data from the pharmacy are logistical records rather than verifiable dispensing records. Therefore, these data may be less accurate than data from an endorsed dispensing database. Thirdly, it is possible that we have overestimated the infants with CHD recommended to receive palivizumab. Patent ductus arteriosus was used to define CHD, however, preterm infants with patent ductus arteriosus would usually not qualify as having CHD as their heart defects are the result of preterm birth. Finally, there could be other factors related to the use of palivizumab that we were unable to include in analyses such as religious or cultural beliefs towards vaccination.

## Conclusion

RSV remains a public health problem and a reason for hospitalisation, especially in high-risk children. This study provides information about the level of palivizumab use in high-risk infants born in WA and shows a varying level of compliance and overall infrequent use. Evidence of incomplete dosing is an important finding in light of recent developments of single dose monoclonal antibodies offering longer protection [25]. Most palivizumab recipients were born preterm and/or had underlying conditions. Understanding the effectiveness of palivizumab in reducing RSV-associated morbidity in the real-world setting, as we recently completed with this dataset, [26] is necessary in order to maximise the protection given by palivizumab.

## Data Availability

We cannot share the individual-level data used for this study under our agreements with the data providers. The datasets analysed during the current study can be applied for from the Western Australian Data Linkage System (http://www.datalinkage-wa.org.au/). Derived data from these datasets are within the paper.

## Abbreviations

ALRI: Acute lower respiratory infection
CHD: Congenital heart disease
CI: Confidence interval
CLD: Chronic lung disease
ICD-10 AM: International Classification of Diseases version 10 Australian modification
NICU: Neonatal intensive care unit
OR: Odds ratio
RSV: Respiratory syncytial virus
WA: Western Australia

## References

[1] Paes BA, Mitchell I, Banerji A, Lanctot KL, Langley JM (2011). A decade of respiratory syncytial virus epidemiology and prophylaxis: Translating evidence into everyday clinical practice. Can Respir J.

[2] Shi T, McAllister DA, O'Brien KL, Simoes EAF, Madhi SA, Gessner BD (2017). Global, regional, and national disease burden estimates of acute lower respiratory infections due to respiratory syncytial virus in young children in 2015: a systematic review and modelling study. Lancet..

[3] Boyce TG, Mellen BG, Mitchel EF, Wright PF, Griffin MR. Rates of hospitalization for respiratory syncytial virus infection among children in medicaid. J Pediatr. 2000;137. 10.1067/mpd.2000.110531 11113845

[4] The IMpact-RSV Study Group (1998). Palivizumab, a humanized respiratory syncytial virus monoclonal antibody, reduces hospitalization from respiratory syncytial virus infection in high-risk infants. Pediatrics..

[5] Sommer C, Resch B, Simoes EA (2011). Risk factors for severe respiratory syncytial virus lower respiratory tract infection. Open Microbiol J.

[6] Ranmuthugala G, Brown L, Lidbury BA (2011). Respiratory syncytial virus--the unrecognised cause of health and economic burden among young children in Australia. Commun Dis Intell.

[7] Moore HC, De Klerk N, Keil AD, Smith DW, Blyth CC, Richmond P (2012). Use of data linkage to investigate the aetiology of acute lower respiratory infection hospitalisations in children. J Paediatr Child Health.

[8] French CE, McKenzie BC, Coope C, Rajanaidu S, Paranthaman K, Pebody R (2016). Risk of nosocomial respiratory syncytial virus infection and effectiveness of control measures to prevent transmission events: a systematic review. Influenza Other Respir Viruses.

[9] Mazur NI, Higgins D, Nunes MC, Melero JA, Langedijk AC, Horsley N (2018). The respiratory syncytial virus vaccine landscape: lessons from the graveyard and promising candidates. Lancet Infect Dis.

[10] American Academy of Pediatrics Committee on Infectious Diseases (2014). Updated guidance for palivizumab prophylaxis among infants and young children at increased risk of hospitalization for respiratory syncytial virus infection. Pediatrics..

[11] The Therapeutic Goods Administration. SYNAGIS palivizumab (rmc) 50 mg / 0.5 mL solution for injection vial 2015 [Accessed: 11 July 2019]. Available rom: http://search.tga.gov.au/s/search.html?collection=tga-artg&profile=record&meta_i=231133.

[12] Western Australian Therapeutic Advisory Group. WATAG Advisory Note: Formulary Status of palivizumab (Synagis) for prophylaxis against respiratory syncytial virus in infants 2012 [Accessed: 20 November 2018]. Available rom: https://ww2.health.wa.gov.au/~/media/Files/Corporate/general%20documents/WATAG/Palivizumab-prophylaxis-for-respiratory-syncitial-virus-in-infants.pdf.

[13] King Edward Memorial Hospital. Neonatal Medication Protocols 2013 [Accessed: 11 July 2019]. Available rom: https://www.kemh.health.wa.gov.au/~/media/Files/Hospitals/WNHS/For%20health%20professionals/Clinical%20guidelines/NCCU/Drug%20Protocols/palivizumab.pdf.

[14] Australian Bureau of Statistics. 2016 Census QuickStats 2018 [accessed: 11 July 2019]. Available rom: https://quickstats.censusdata.abs.gov.au/census_services/getproduct/census/2016/quickstat/036.

[15] Chow SSW, Le Marsney R, Hossain S, Haslam R, Liu K (2015). Report of the Australian and New Zealand neonatal network.

[16] Holman CD, Bass AJ, Rosman DL, Smith MB, Semmens JB, Glasson EJ (2008). A decade of data linkage in Western Australia: strategic design, applications and benefits of the WA data linkage system. Aust Health Rev.

[17] Christenen D, Davis G, Draper G, Mitrou F, McKeown S, Lawrence D (2014). Evidence for the use of an algorithm in resolving inconsistent and missing indigenous status in administrative data collections. Aust J Soc Issues.

[18] Pink B. Information paper: an introduction to socio-economic indexes for areas (SEIFA), 2006. Australian Bureau of Statistics; 2008.

[19] Moore HC, Keil AD, Richmond PC, Lehmann D (2009). Timing of bronchiolitis hospitalisations and respiratory syncytial virus immunoprophylaxis in non-metropolitan Western Australia. Med J Aust.

[20] Trist S, Horsley E, Katf H, Tasker N, Mostaghim M. Improving the prescribing of palivizumab. J Paediatr Child Health. 2018. 10.1111/jpc.14083 29863814

[21] Frogel M, Stewart D, Hoopes M, Fernandes A, Mahadevia P (2010). A systematic review of compliance with palivizumab administration for RSV immunoprophylaxis. J Manag Care Pharm.

[22] Stewart DL, Ryan KJ, Seare JG, Pinsky B, Becker L, Frogel M (2013). Association of RSV-related hospitalization and non-compliance with Palivizumab among commercially insured infants: a retrospective claims analysis. BMC Infect Dis.

[23] Golombek SG, Berning F, Lagamma EF (2004). Compliance with prophylaxis for respiratory syncytial virus infection in a home setting. Pediatr Infect Dis J.

[24] Moore H, Burgner D, Carville K, Jacoby P, Richmond P, Lehmann D (2007). Diverging trends for lower respiratory infections in non-Aboriginal and Aboriginal children. J Paediatr Child Health.

[25] Domachowske JB, Khan A, Esser MT, Jensen KM, Takas T, Villafana T (2017). A single dose monoclonal antibody (mAb) immunoprophylaxis strategy to prevent RSV disease in all infants: results of the first in infant study with MEDI8897. Open forum infectious diseases.

[26] Moore HC, de Klerk N, Richmond PC, Fathima P, Xu R, Keil AD, et al. Effectiveness of Palivizumab against respiratory syncytial virus: cohort and case series analysis. J Pediatr 2019;214:121–127 e1. 10.1016/j.jpeds.2019.06.058 31378522

